# Binary and Ternary Inclusion Complexes of Niflumic Acid: Synthesis, Characterization, and Dissolution Profile

**DOI:** 10.3390/pharmaceutics16091190

**Published:** 2024-09-09

**Authors:** Zohra Bouchekhou, Amel Hadj Ziane-Zafour, Florentina Geanina Lupascu, Bianca-Ștefania Profire, Alina Nicolescu, Denisse-Iulia Bostiog, Florica Doroftei, Ioan-Andrei Dascalu, Cristian-Dragoș Varganici, Mariana Pinteala, Lenuta Profire, Tudor Pinteala, Bachir Bouzid

**Affiliations:** 1 Chemical Engineering Laboratory, Process Engineering Department, Faculty of Technology, University of Blida 1, Road of Soumaa, BP 270, Blida 09000, Algeria; bouchekhou_zohra@univ-blida.dz (Z.B.); hadj.ziane2020@gmail.com (A.H.Z.-Z.); drbouzid@gmail.com (B.B.); 2 Department of Pharmaceutical Chemistry, Faculty of Pharmacy, “Grigore T. Popa” University of Medicine and Pharmacy of Iași, 16 Universitaty Street, 700115 Iași, Romania; florentina-geanina.lupascu@umfiasi.ro; 3 Department of Internal Medicine, Faculty of Medicine, “Grigore T. Popa” University of Medicine and Pharmacy of Iasi, 16 University Street, 700115 Iași, Romania; bianca-stefania.profire@umfiasi.ro; 4 “Petru Poni” Institute of Macromolecular Chemistry, 41A Grigore Ghica-Voda Alley, 700487 Iasi, Romania; alina@icmpp.ro (A.N.); bostiog.denisse@icmpp.ro (D.-I.B.); florica.doroftei@icmpp.ro (F.D.); idascalu@icmpp.ro (I.-A.D.); varganici.cristian@icmpp.ro (C.-D.V.); pinteala@icmpp.ro (M.P.); 5 Department of Orthopedics and Traumatology, Faculty of Medicine, “Grigore T. Popa” University of Medicine and Pharmacy of Iasi, 16 University Street, 700115 Iași, Romania; tudor_pinteala@umfiasi.ro

**Keywords:** niflumic acid, 2-hydroxypropyl-β-cyclodextrin, inclusion complex, complexation efficiency, drug delivery, solubility, dissolution profile

## Abstract

Although niflumic acid (NA) is one of the most used non-steroidal anti-inflammatory drugs, it suffers from poor solubility, low bioavailability, and significant adverse effects. To address these limitations, the complexation of NA with cyclodextrins (CDs) is a promising strategy. However, complexing CDs with low molecular weight drugs like NA can lead to low CE. This study explores the development of inclusion complexes of NA with 2-hydroxypropyl-β-cyclodextrin (2HP-β-CD), including the effect of converting NA to its sodium salt (NAs) and adding hydroxypropyl methylcellulose (HPMC) on complex formation. Inclusion complexes were prepared using co-evaporation solvent and freeze-drying methods, and their CE and Ks were determined through a phase solubility study. The complexes were characterized using physicochemical analyses, including FT-IR, DSC, SEM, XRD, DLS, UV-Vis, ^1^H-NMR, and ^1^H-ROESY. The dissolution profiles of the complexes were also evaluated. The analyses confirmed complex formation for all systems, demonstrating drug–cyclodextrin interactions, amorphous drug states, morphological changes, and improved solubility and dissolution profiles. The NAs-2HP-β-CD-HPMC complex exhibited the highest CE and Ks values, a 1:1 host-guest molar ratio, and the best dissolution profile. The results indicate that the NAs-2HP-β-CD-HPMC complex has potential for delivering NA, which might enhance its therapeutic effectiveness and minimize side effects.

## 1. Introduction

The improvement of the pharmacokinetic profile of classical drugs using modern pharmaceutical formulations, such as controlled drug delivery systems (CDDSs), is an important research field because it is less expensive than developing new drugs [1]. CDDSs allow for a longer period of drug release, hence reducing the frequency at which the drug has to be taken. These formulations may be roughly categorized into the following three primary types based on their evolution [2]: (i) first-generation formulations were designed to control the physiochemical characteristics of the drugs by using dissolution, diffusion, or osmosis and are typically administered once or twice a day; (ii) second-generation formulations aimed to deliver the drug over a longer period with better control using a zero-order release mechanism, but it still has limited targeting capabilities for specific organs; (iii) third-generation formulations focus on administering the drug without invasive methods, such as targeted drug delivery that bypasses the blood–brain barrier, but it still faces challenges in overcoming physiological and biological barriers. 

To achieve optimal pharmacodynamic properties, such as a rapid onset of action [3], many studies have been conducted to improve drugs’ solubilities and dissolution rates. These strategies include reducing the particle size or crystallinity of the drug [4,5], preparing microspheres [6], formulating cocrystals, and using polymer carriers [7]. In addition, the oral route is the most preferred method of drug administration due to its cost-effectiveness, favorable outcomes, and safety for patients compared to other forms of treatment [8]. However, when drugs are orally administered, they are prone to quick denaturation and degradation and must overcome many obstacles before reaching the desired target, resulting in low drug bioavailability [9].

One effective method to overcome these drawbacks is complexation with cyclodextrins (CDs). CDs are cyclic oligosaccharides with six (α-CD), seven (β-CD), and eight (γ-CD) glucopyranoside units linked by α-1,4-glycosidic bonds, with hydrophilic moiety at the surface and hydrophobic moiety at the center [10]. The torus shape of CDs allows them to form inclusion complexes with several drugs through non-covalent interactions like hydrogen bonds and van der Waals forces [11,12]. 

CD-based inclusion complexes offer several advantages over other polymer carriers, such as micelles and vesicles [13]. Unlike polymer carriers, CDs enhance drug solubility, stability, and bioavailability, requiring versatile formulation processes. Additionally, CDs provide protection against the degradation of drugs, offer high flexibility for drug release profiles through chemical modifications, and are generally non-toxic [14]. 

In order to enhance the solubility and stability of β-CD, making it more suitable for pharmaceutical applications, chemically modified forms of β-CD were synthesized. For example, 2-hydroxypropyl-β-cyclodextrin (2HP-β-CD) is a β-CD derivative, in which hydroxyl groups of the C6 atom of the glucose units are replaced with the 2-hydroxypropyl group [15]. It is recognized as safe and well-tolerated by the US FDA [16], being widely used to improve drugs’ solubility, dissolution rate, and chemical stability and reduce the gastrointestinal side effects of drugs [17,18,19]. 

It is known that drugs with molecular weights between 200 and 400 Da and CDs with molecular weights between 1200 and 1500 Da often result in low CE [20,21]. As a result, numerous studies have been conducted to enhance the CE of drugs into CDs using different methods, such as salt formation, adding water-soluble polymers, charge–charge interactions, multiple complexations, and metal complexes [22]. In this context, the addition of small amounts of hydrophilic polymers [23] has demonstrated significant potential, including a reduction in the number of CDs used for complexation [24], acting as film-forming agents, enteric film-coated materials, and matrix-forming agents [2]. Moreover, using synergistic excipients can increase the CE and stability of the system and adjust the desired drug release kinetics from CDDSs [25,26,27].

Hydroxypropyl methylcellulose (HPMC) is a hydrophilic polymer often used to generate controlled and sustained-release formulations for many oral drugs due to its mucoadhesive properties through a longer residence duration in the gastrointestinal tract (HPMC is chemically stable at a pH range of 3 to 1), biocompatibility, biodegradability, and swelling ability [15]. Furthermore, HPMC promotes cellular permeability because of its numerous hydroxyl groups, which allow HPMC to adhere to negatively charged cell membranes via electrostatic or hydrogen bonds [8].

Niflumic acid (NA) (2-[[3-(trifluoromethyl)-phenyl]-amino]-3-pyridine carboxylic acid) is a non-steroidal anti-inflammatory drug (NSAID) known for its anti-inflammatory, analgesic, and antipyretic properties [28]. It provides significant benefits for relieving acute pain and treating rheumatoid arthritis, arthrosis, and other joint diseases [29]. According to the Biopharmaceutics Classification System (BCS), NA is categorized as a class II compound, characterized by its poor water solubility (26 μg/mL at 25 °C), lipophilicity, and high permeability [30]. Additionally, the maximum concentration of NA varied widely, ranging from 188 to 4121 ng/mL, depending on the analytical methods used [31,32]. These properties contribute to its limited dissolution rate and poor oral bioavailability, thereby impacting its therapeutic efficacy. It also induces several side effects, such as gastrointestinal irritation and ulceration, renal insufficiency, hepatotoxicity, and cutaneous reactions, along with poor selectivity between normal and inflamed tissues [33]. 

In order to improve the physicochemical and pharmacokinetic profile and pharmacological effects of NA, several studies focused on NA-based CD inclusion complexes were reported [34,35,36]. Given that the molecular weight of NA is approximately 282.22 Da, the CE of NA into CD derivatives like 2HP-β-CD could be reduced. Referring to NA-2HP-β-CD, there is limited research focused on simultaneously optimizing inclusion complex formation and maintaining stability. The combination of two approaches, such as salt formation and adding hydrophilic polymers, could effectively enhance both the CE and apparent stability [37,38]. 

The main goal of our study was to evaluate the synergistic effect of 2HP-β-CD and HPMC, as well as the salt formation on the pharmacokinetic profile of NA, focusing on key parameters, such as complexation efficiency (CE) and the stability constant (Ks) of the resulting complex. More specifically, binary and ternary inclusion complexes based on NA and NAs were synthesized and studied in terms of the CE, solubility, physicochemical properties, and dissolution rate of the drug. This objective is accomplished through a comparative study aimed at identifying the most effective formulation for optimizing NA delivery. The physicochemical properties of these complexes in the solid and liquid states were evaluated using FT-IR, DSC, XRD, SEM, DLS, UV-Vis, and ^1^HNMR spectroscopy. Furthermore, the phase solubility and in vitro dissolution profiles were assessed

## 2. Materials and Methods

### 2.1. Materials

Niflumic acid (NA) was a kind gift from Saidal Group (Oued Smar, Algiers, Algeria); 2-hydroxypropyl-β-cyclodextrin (2HP-β-CD) with a molar substitution of 0.8 hydroxypropyl groups per glucopyranose unit and a molecular weight of ~1460 Da (hydroxypropyl)methylcellulose (HPMC), with a molecular weight of 86 kDa, was purchased from Sigma Aldrich (Steinheim, Germany). All remaining chemicals and solvents were of analytical grade and were used without additional purification.

### 2.2. Phase Solubility Studies

Phase solubility studies were conducted for both binary and ternary complexes according to the method described by Higuchi and Connors [39]. In a series of 25 mL volumetric flasks containing different concentrations of 2HP-β-CD (ranging from 0.003 to 0.015 M) in the presence and absence of 50 mg of HPMC (0.2% (*w*/*v*)), 25 mL of distilled water (pH 7.4) mixed with an excess amount of NA (200 mg) was added. Similarly, the phase solubility study for NAs was conducted, in which excess amounts of NAs (200 mg) were added to aqueous solutions containing increasing concentrations of 2HP-β-CD (0.002 to 0.009 M) in the presence and absence of 50 mg HPMC (0.2% *w*/*v*). The suspensions were then mechanically shaken at 300 rpm (Heidolph Titramax 100, Heidolph, Hamburg, Germany) for 72 h at room temperature (25 °C) until equilibrium was reached. After equilibration, the solutions were filtered (Cloup syringe filter, PVDF, 0.22 µm pore size) and diluted accordingly (2 mL in 25 mL of distilled water). The drug concentration in each solution was determined spectrophotometrically (UV-Vis Cintra 2020, GBC Scientific Equipment, Keysborough, Australia) at 289 nm. Each experiment was performed in triplicate.

The apparent stability constants (Ks) and the complexation efficiency (CE) were calculated from the slope of the phase solubility diagrams using Equations (1) and (2) [40,41], as follows:

Ks = Slope/S_0_(1 − slope) (1)

CE = Slope/(1 − slope)(2)

where S_0_ represents the solubility of the drug in water in the absence of 2HP-β-CD (for the binary system) or 2HP-β-CD and HPMC (for the ternary system). The slope was obtained from the plot of NA and NAs concentration against 2HP-β-CD and 2HP-β-CD-HPMC, respectively.

### 2.3. Preparation of the Inclusion Complexes

#### 2.3.1. Preparation of NA Binary and Ternary Inclusion Complexes

Binary (NA-2HP-β-CD) and ternary (NA-2HP-β-CD-HPMC) systems were prepared using a combination of the following two methods: solvent co-evaporation and freeze-drying [42,43]. To achieve a 1:1 molar ratio of the drug and CD, an aqueous phase containing a solution of 2HP-β-CD (0.012 mol/L) in 15 mL of distilled water, with and without an optimized quantity of HPMC (0.2%, *w*/*v*), was combined with an organic phase containing a solution of NA (0.088 mol/L) in 2 mL of ethanol. This ratio has been identified as optimal in the literature [35,44]. The aqueous and organic phases were stirred and heated to 50 °C for 2 h. Next, the ethanol was removed under vacuum using a rotary evaporator (Büchi, R-215, Flawil, Switzerland). The resulting suspensions were filtered using membrane filters (Merk Millipore, Burlington, MA, USA) with a pore size of 0.22 µm. The filtrate was then frozen at −20 °C and freeze-dried for 10 h using a lyophilizer (Christ Alpha 1-2LD Plus, Martin Christ, Osterode am Harz, Germany). The resulting complexes were powdered and stored in a desiccator for further analysis.

#### 2.3.2. Preparation of the NAs’ Binary and Ternary Inclusion Complexes

NA was converted to its sodium salt by adding 4.65 g (0.01 mol) of NA to an aqueous solution containing 0.02 mol of NaOH. The resulting suspension was boiled and filtered while it was still hot. The filtrate was then refrigerated until precipitation occurred, and the resulting solution was evaporated to obtain dry NAs [45].

The NAs-based systems (NAs-2HP-β-CD and NAs-2HP-β-CD-HPMC) were prepared using the same method as corresponding NA-based systems (NA-2HP-β-CD) NA-2HP-β-CD-HPMC).

### 2.4. Drug Content Quantification

The content of the drug (NA, NAs) in the complexes was determined using solvent extraction. Each complex (10 mg) was dissolved in ethanol in a 10 mL volumetric flask, stirred overnight, and sonicated for 15 min. The solution was then adjusted to the desired volume (10 mL) with ethanol, filtered through a 0.22 µm Cloup syringe filter, and appropriately diluted (2 mL in 10 mL of ethanol for NA binary and ternary complex and 0.5 mL in 10 mL of ethanol for NAs ternary complex). The solutions were analyzed using UV-Vis spectroscopy at 289 nm. The extraction was performed in triplicate, and the drug content of each sample was calculated using Equation (3) [46], as follows:

The drug content (%) = (mass of extracted drug/mass of complex) × 100(3)

### 2.5. Physicochemical Characterization of the Inclusion Complexes

#### 2.5.1. Ultraviolet-Visible Spectroscopy (UV-Vis)

A UV-Vis spectrophotometric analysis was performed using a Cintra 2020 (GBC Scientific Equipment, Keysborough, Australia) UV-Vis spectrophotometer with 1.0 cm quartz cells. The wavelength of 289 nm was selected for the quantification of NA. The presence of 2HP-β-CD and HPMC did not interfere with the spectrophotometric assay of the drug. 

#### 2.5.2. Fourier Transform Infrared Spectroscopy (FT-IR)

The samples were assessed using the FT-IR method by evaluating changes in peak shape, position, and intensity. The FT-IR spectra of the samples were obtained using an ABB MB3000 FT-IR (ABB, Québec, QC, Canada) analyzer in the wave number range of 4000–650 cm^−1^, with a resolution of 2 cm^−1^ in transmission mode at ambient temperature.

#### 2.5.3. Differential Scanning Calorimetry (DSC)

The DSC curves were recorded using a DSC 200 F3 Maia instrument (Netzsch, Waldkraiburg, Germany). A total of 5.5 mg of each sample was added to aluminum crucibles, which were then sealed with pierced lids. The samples were heated at a rate of 10 °C/min under a nitrogen flow rate of 50 mL/min, which served as an inert working atmosphere.

#### 2.5.4. Scanning Electronic Microscopy (SEM) and X-ray Powder Diffraction (XRD)

The particle morphology and crystalline structure of NA, 2HP-β-CD, HPMC, and corresponding inclusion complexes were investigated using a Verios G4 UC scanning electron microscope (Thermo Fisher Scientific, Brno-Černovice, Czech Republic) equipped with an energy-dispersive X-ray spectroscopy analyzer (Octane Elect Super SDD detector, Pleasanton, CA, USA). For SEM examination, the samples were fixed on aluminum stubs with double-adhesive carbon tape and coated with 6 nm platinum using a Leica EM ACE200 sputter coater (Leica, Vienna, Austria) to provide electrical conductivity and prevent charge accumulation during electron beam exposure. The morphological study was carried out using a secondary electron detector (ETD detector—Everhart–Thornley detector) to highlight the shape and size of the particles. The SEM micrographs were made using an acceleration voltage of 5 kV and a spot size of 0.4 nA. For X-ray powder analysis, the samples were analyzed with a 2θ angle range of 2–50° and a scan rate of 1°/min, with a step size of 0.01.

#### 2.5.5. Dynamic Light Scattering (DLS) 

The hydrodynamic diameter of the lyophilized complexes and their components were measured using a flow cell module on a Delsa Nano C Submicron Particle Size Analyzer (Beckman Coulter, Brea, CA, USA). Prior to the measurements, the suspensions were prepared by resuspending the complexes and their components in deionized water. Each measurement was performed in triplicate in a 5 mL cuvette.

#### 2.5.6. Nuclear Magnetic Resonance Spectroscopy (NMR)

NMR spectroscopy is a crucial tool for confirming the inclusion phenomenon of a guest drug molecule within a host CD molecule. The ^1^H-NMR spectra and 2D ^1^H,^1^H-ROESY spectra were recorded using a 600 MHz Bruker Avance NEO spectrometer (Bruker Biospin, Ettlingen, Germany) equipped with a 5 mm inverse detection z-gradient probe. The NMR spectra were acquired at 27 °C. For the NMR analysis, the inclusion complexes and their components were dissolved in D_2_O. The chemical shift values (δ) were reported in ppm.

### 2.6. In Vitro Dissolution Studies

Dissolution studies for NA and its complexes were performed using the United States Pharmacopoeia Paddle Method (Apparatus II) [47], with some modifications on a Distek Dissolution System 2500 (Distek, Inc., North Brunswick, NJ, USA). A total of 0.7 mg of NA and an equivalent amount of each complex, which contained 0.7 mg of NA, were added to 75 mL of 0.1 M phosphate buffer (pH 6.8) as the dissolution medium. The medium was maintained at 37 ± 0.5 °C and stirred at 100 rpm. A total of 5 mL of solution was withdrawn by means of a syringe at time intervals of 2, 5, 10, 15, 20, 30, 45, and 60 min. The sample was then filtered through a 0.22 µm syringe filter and analyzed immediately using a UV-Vis spectrophotometer at 289 nm. Each experiment was performed in triplicate. The dissolution profiles were created by plotting the cumulative percentage of the drug released over time.

In addition, several mathematical models were applied in order to analyze the experimental data. These models, including the zero-order, first-order, Higuchi, Hixson–Crowell, and Korsmeyer–Peppas models, are reported in the literature for their effectiveness and applicability for kinetic profile studies [48].

### 2.7. Statistical Analysis

A statistical analysis was performed using OriginPro 2024 (OriginLab, Northampton, MA, USA), the data were expressed as the mean value ± standard deviation (SD) of three independent experiments. The findings were compared and assessed statistically using one-way analysis of variance (ANOVA) with Tukey’s test, with *p* < 0.05 considered significant.

## 3. Results and Discussions

### 3.1. Phase Solubility Studies

**Figure 1:**
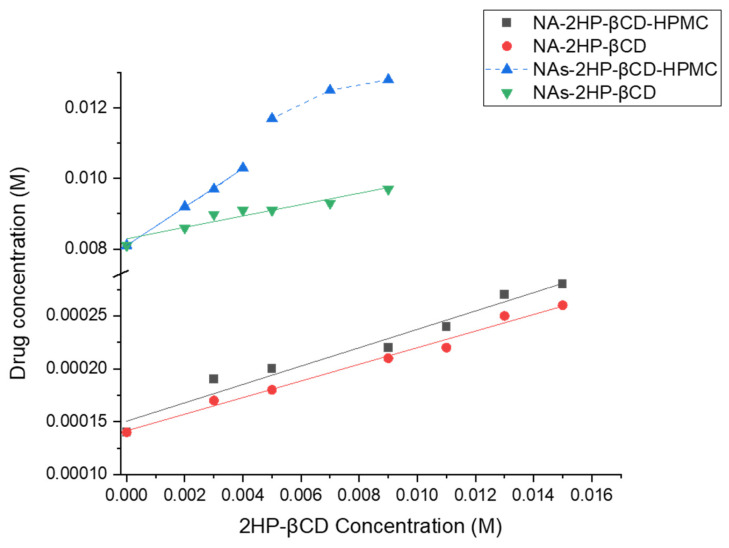
Phase solubility diagrams of the binary (NA/NAs-2HP-β-CD) and ternary (NA/NAs-2HP-β-CD-HPMC) systems. The lines represent the best-fit linear regression of the data points (n = 3, coefficient of variation < 3%, error bars not shown for clarity).

The phase solubility diagram for binary (NA/NAs-2HP-β-CD) and ternary (NA/NAs-2HP-β-CD-HPMC) systems shows a linear increase in drug solubility (Figure 1). Also, according to Higuchi and Connors [39], NA/NAs-2HP-β-CD and NA-2HP-β-CD-HPMC are type-A_L_ complexes, while the NAs-2HP-β-CD-HPMC system can be classified as a type-A_N_ system. It was observed that the aqueous solubility of the complexes increased linearly with increasing 2HP-β-CD concentrations. This is likely due to the formation of the inclusion complex through hydrophobic interactions between the drug and the walls of the 2HP-β-CD macrocyclic cavity [35]. 

However, in the concentration range from 0.005 to 0.009 M of 2HP-β-CD, a deviation from linearity occurs in the NAs-2HP-β-CD-HPMC complex. This may be associated with ligand-induced changes in the dielectric constant of the solvent, changes in the physical properties of the solution, or the self-association of the ligands at high CD concentrations [4]. This observation indicates that at higher concentrations, CD is less effective [49,50]. Consequently, a reduced quantity of CD is required to formulate the NAs-2HP-β-CD-HPMC complex, resulting in economic advantages. The phase solubility data are presented in Table 1. 

It has been reported that hydrophilic polymers can increase the Kc and CE of the complex and improve the aqueous solubility of the drug when used at low concentrations [51]. In our experiment, it was observed that when HPMC was added, the Ks and CE values were increased in both the binary and ternary systems, with more intense improvement being recorded in the case of NAs-2HP-β-CD-HPMC. In the case of NAs-2HP-β-CD-HPMC, the value recorded for Ks and CE was approximately 6.3-fold higher than that of NAs-2HP-β-CD (Table 1). Converting NA into NAs also resulted in a more notable influence of Ks and CE. The CE was increased in both binary and ternary systems, with the increase being 24-fold in the case of NAs-2HP-β-CD vs. NA-2HP-β-CD binary and approximately 140-fold in the case of NAs-2HP-β-CD-HPMC vs. NA-2HP-β-CD-HPMC ternary systems. This improvement is attributed to the increased aqueous solubility of NAs compared to NA drugs, which facilitated the interaction with CD and consequently enhanced the CE.

More interesting data were obtained referring to Ks values. In the case of NAs-2HP-β-CD, Ks was 2.3-fold less than NA-2HP-β-CD, which means that NAs could destabilize the complex in an aqueous solution. Similar data were obtained by Yesook et al., who found that ziprasidone salt decreases the apparent Ks while increasing its CE [52]. At the same time, in the case of NAs-2HP-β-CD-HPMC, the value recorded for Ks was approximately threefold higher than NAs-2HP-β-CD-HPMC, which supports the favorable influence of HPMC on the stability of the systems and the drug’s complexation efficiency. 

If we discuss the phase solubility profile, in terms of Ks and CE, of NAs-2HP-β-CD-HPMC vs. NA-2HP-β-CD, we can observe the synergic effect of NAs and HPMC, with the values recorded for NAs-2HP-β-CD-HPMC being approximately 3-fold (Ks) and 150-fold (CE) higher than NA-2HP-β-CD. 

It was reported that complexes with a Ks between 100 and 1000 M^−1^ are more stable and proper for biological applications, while the complexes with a Ks lower than 100 M^−1^ are highly unstable, and those with a Ks higher than 1000 M^−1^ could negatively impact drug absorption [53]. Based on our results, we can conclude that NAs-2HP-β-CD-HPMC is the most stable complex, while NA/NAs-2HP-β-CD and NA-2HP-β-CD-HPMC are unstable systems (Table 1). 

Although these findings pertain to the liquid state, future studies are essential to evaluate the complex’s stability in the solid state, which is a critical factor in assessing the long-term performance of the complexes. Such studies will also aid in determining the complex’s behavior under recommended storage conditions and establishing its shelf life.

### 3.2. Drug Content Quantification

The drug content (%) and the amount of the drug in the 10 mg complex were calculated (Table 2). The drug content (%) value of ternary systems (NA/NAs-2HP-β-CD-HPMC) was higher than the value recorded for the NA-2HP-β-CD complex. The highest value was recorded for NAs-2HP-β-CD-HPMC, which was 18.5-fold higher than that of NA-2HP-β-CD and 9.6-fold higher than that of NA-2HP-β-CD-HPMC. These data strongly support the efficiency of the NAs-based ternary complex. 

### 3.3. Physicochemical Characterization of the Inclusion Complexes

#### 3.3.1. Fourier Transform Infrared Spectroscopy (FT-IR)

**Figure 2:**
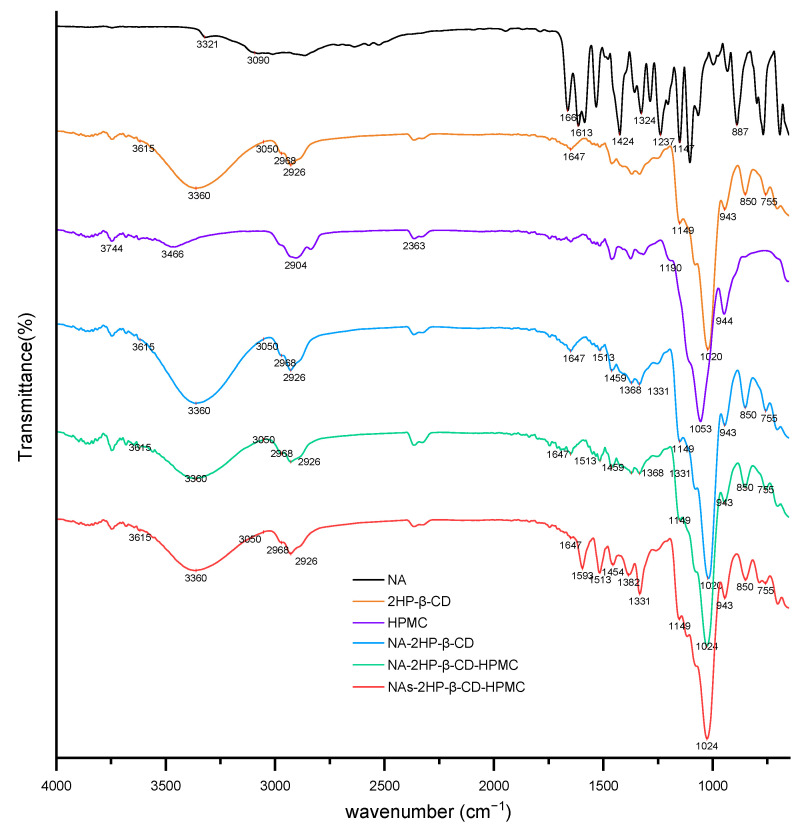
FT-IR spectra of NA, 2HP-β-CD, HPMC, binary (NA-2HP-β-CD), and ternary (NA/NAs-2HP-β-CD-HPMC) systems.

Infrared spectroscopy is commonly used to characterize the molecular interactions between host and guest molecules. After complexation, the absorption bands or intensities of certain functional groups may change when the guest drug molecules are encapsulated in the CD cavity [54]. Figure 2 shows the FT-IR spectra of NA, 2HP-β-CD, HPMC, and the inclusion complexes. For NA, the spectrum showed an absorption peak at 3321 cm^−1^ (N–H stretching vibration) and a broad signal at 3090 cm^−1^ (C–H stretching vibration from the benzene ring). Major characteristic peaks were observed at 1661 cm^−1^ (C=O stretching), 1613 cm^−1^ (primary amine), 1424 cm^−1^ (O–H stretching), 1326 cm^−1^ (CF_3_ group signal), 1237 cm^−1^ and 1147 cm^−1^ (C–N aliphatic and aromatic bands), and 887 cm^−1^ (C–H band) [55]. 

HPMC was characterized by a main peak at 1053 cm^−1^ (C–O–C stretching). The 2HP-β-CD spectrum showed a broad band between 3050 cm^−1^ and 3615 cm^−1^ (O–H stretching vibration), the bands from 2968 cm^−1^ and 2926 cm^−1^ (C–H stretching), the peaks at 1647 cm^−1^ (H–O–H bending) and 1149 cm^−1^ (C–O stretching), and the peak at 1020 cm^−1^ (C–O–C stretching). Absorption peaks at 943 cm^−1^, 850 cm^−1^, and 755 cm^−1^ are specific to CDs and are attributed to backbone vibration due to α-1,4-glycosidic bonds, anomeric CH deformation, and pyranose ring vibration, respectively [56]. 

The inclusion complexes spectra showed a notable decrease in the peak intensity of NA, and shifts were observed, which indicates that a host–guest interaction has occurred and confirms the formation of the NA-based inclusion complex [57]. In the case of NA-based complex (NA-2HP-β-CD and NA-2HP-β-CD-HPMC complexes) spectra, the NA peaks were shifted to 1513 cm^−1^, 1459 cm^−1^, 1368 cm^−1^, and 1331 cm^−1^. The spectrum of NAs-2HP-β-CD-HPMC showed more significant changes, indicating the formation of a more efficient and amorphous complex [58,59]. Therefore, in the NAs-2HP-β-CD-HPMC spectrum, the characteristic peaks of NA at 1661 cm^−1^, 1613 cm^−1^, 1424 cm^−1^, 1326 cm^−1^, and 1237 cm^−1^ were shifted to 1593 cm^−1^, 1513 cm^−1^, 1454 cm^−1^, 1382 cm^−1^, and 1331 cm^−1^.

#### 3.3.2. Differential Scanning Calorimetry (DSC)

**Figure 3:**
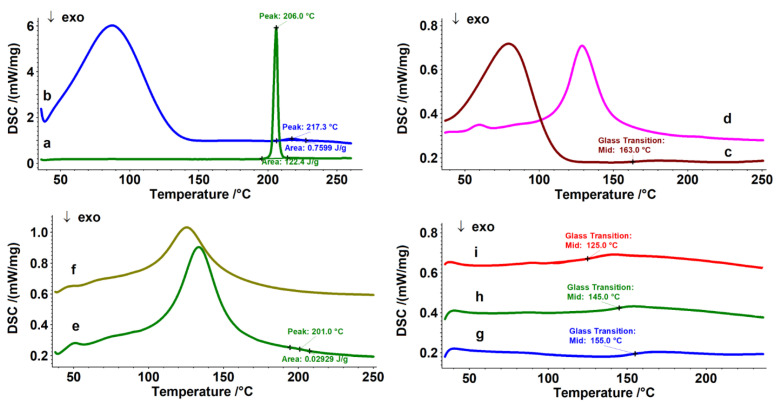
DSC curves of the studied structures: NA (a); NA-2HP-β-CD (b); HPMC (c) (first heating); 2HP-β-CD (d); NA-2HP-β-CD-HPMC (e); NAs-2HP-β-CD-HPMC (f); HPMC (g) (second heating); NA-2HP-β-CD-HPMC (h) (second heating); and NAs-2HP-β-CD-HPMC (i) (second heating).

DSC is a method used to record the heat flow vs. temperature for a wide range of materials, including but not limited to polymers. It provides crucial information about solid-state interactions [60,61,62,63]. In this study, we used DSC to examine the interaction between host and guest molecules. When drug molecules are included in the CD cavity, their melting points usually shift to different temperatures or disappear [64]. Figure 3 shows the DSC curves of the studied inclusion complexes. The broad endothermic profiles up to approximately 180 °C (curves b–f) represent the loss of physical and/or crystallized water from the host molecule 2HP-β-CD and HPMC, respectively. The guest molecule, pure NA, displayed a sharp and intense melting profile at 206 °C, which is in accordance with the literature [14], with an enthalpy (ΔH_NA_) value of 122.4 J g^−1^ (curve a). Upon analyzing the DSC curve of the binary inclusion complex NA-2HP-β-CD, it is evident that the melting profile of NA significantly decreases in intensity (ΔH_NA-2HP-β-CD_ = 0.7599 J g^−1^) and shifts to a higher temperature by more than 10 °C (217 °C) (curve b). This suggests the formation of a new solid amorphous phase through complexation [65].

The same aspect may be observed for the ternary inclusion complexes NA/NAs-2HP-β-CD-HPMC, in which the melting profile of NA almost disappeared (ΔH_NA-2HP-β-CD-HPMC_ = 0.02929 J g^−1^), with its value displaced to 201 °C for NA-2HP-β-CD-HPMC (curve e) and completely disappearing for NAs-2HP-β-CD-HPMC, respectively (curve f), which could be proof of the formation of the ternary inclusion complex [66]. Similar results were reported by Grebogi et al. [67].

The melting profile of the guest molecule (NA, NAs) in inclusion complexes decreased and disappeared due to the thermal protection provided by the cavity in which the drug is entrapped [68,69,70].

Curve c from Figure 3, shows that the pristine HPMC exhibited a glass transition temperature domain (Tg) at 163 °C on the first heating run [71]. To best highlight Tg, a second heating run was conducted on all structures containing HPMC in order to eliminate any previous thermal history (curves g–i). The pristine HPMC has a Tg of 155 °C (curve g). It is a known fact that the Tg is a transition consistent with the amorphous phase, and its degree increases with a decrease in Tg. From curve i, it can be seen that the Tg of HPMC in NA-2HP-β-CD-HPMC (curve h) has decreased by 10 °C, from 155 °C to 145 °C, and curve i showed a significant decrease in Tg for NAs-2HP-β-CD-HPMC, from 155 °C to 125 °C.

#### 3.3.3. Scanning Electronic Microscopy (SEM) and X-ray Powder Diffraction (XRD)

**Figure 4:**
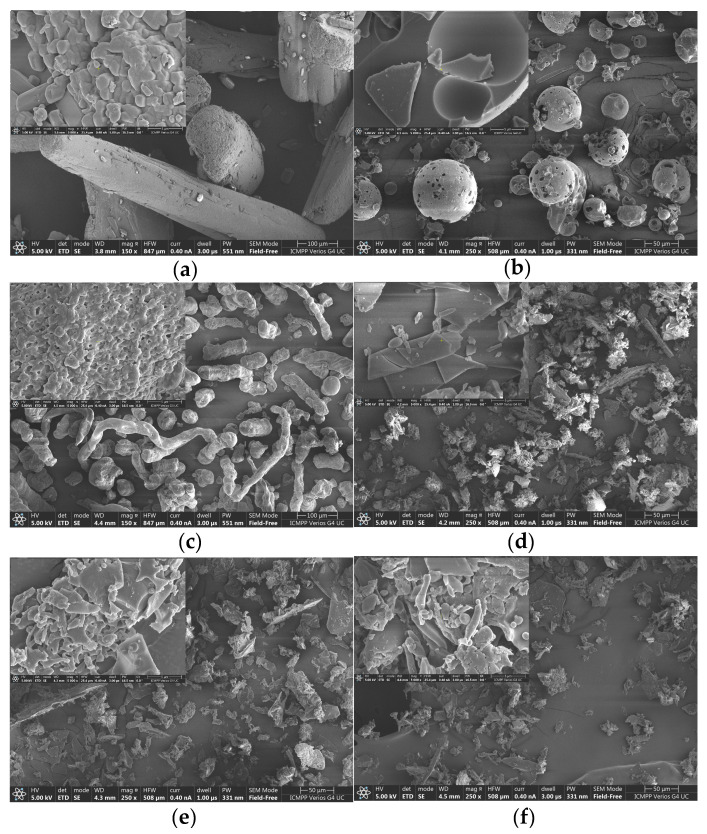
SEM micrographs of NA (**a**), 2HP-β-CD (**b**), HPMC (**c**), NA-2HP-β-CD (**d**), NA-2HP-β-CD-HPMC (**e**), and NAs-2HP-β-CD-HPMC (**f**).

In this study, SEM is used to characterize the surface morphology of the pristine materials (NA, 2HP-β-CD, and HPMC) and NA-/NAs-based inclusion complexes (Figure 4). Although this method alone may not conclusively demonstrate the formation of true inclusion complexes, the captured images serve to verify the structural changes that occur after complex formation and act as key indicators for predicting their formation [35]. The following two magnifications were used to capture the micrographs: 250×, except for NA and HPMC, which were captured at 150× to highlight sample homogeneity, and 5000× to highlight morphological details. Pure NA manifested as long cylindrical crystals of irregular size, whereas 2HP-β-CD particles appeared amorphous, spherical, and perforated, and HPMC showed elongated, crumpled, and thin fibers. All inclusion complexes showed morphological changes, exhibiting distinct, amorphous, irregular structures and bulk agglomerates. Notably, strong morphological similarities were observed between the binary and ternary systems, with only minor differences observed in the ternary complexes. In particular, HPMC was observed to be adsorbed on the surface in micrographs magnified at 5000×. This is in agreement with other studies indicating that HPMC cannot be included in the CD cavity because its effective diameter is higher than that of the CD cavity [72]. In this case, intramolecular hydrogen bonds between CD and HPMC could occur. These findings are consistent with the X-ray diffraction results and provide compelling evidence for the formation of inclusion complexes. 

**Figure 5:**
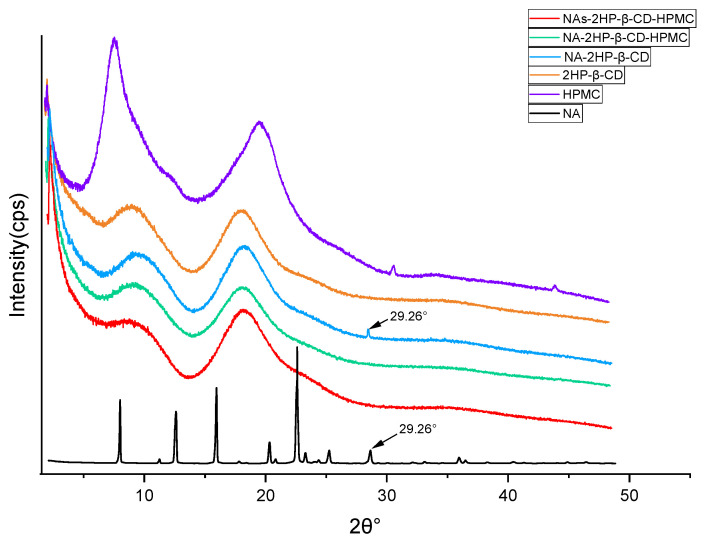
XRD patterns of NA, 2HP-β-CD, HPMC, NA-2HP-β-CD, NA-2HP-β-CD-HPMC, and NAs-2HP-β-CD-HPMC.

X-ray diffraction analysis is essential to confirm the formation of supramolecular complexes. It is used to compare the diffraction patterns of the drug and its complexes; if optimal complexes have been obtained, these diffraction patterns must be clearly distinct [66]. NA showed high-intensity peaks at the following specific 2θ values: 08.06°, 12.79°, 16.22°, 20.72°, 23.06°, 25.77°, and 29.26°, indicating drug crystallinity (Figure 5); these values are in agreement with those documented by Radacsi et al. [73]. In contrast, the diffractograms of 2HP-β-CD and HPMC revealed two broad halos, suggesting an amorphous state. A hollow pattern, similar to that of the pure 2HP-β-CD, was also recorded for the inclusion complexes (Figure 5) without any distinct peaks, except for the binary complex NA-2HP-β-CD, which shows a peak characteristic of NA at a 2θ value of 29.26°, indicating an incomplete complexation between NA and 2HP-β-CD. For the ternary complexes, the absence of any peaks corresponding to NA indicated the formation of a true inclusion complex, this result being in full agreement with the DSC analysis. On the other hand, a comparison between the patterns of the pure NA and the inclusion complexes indicates the amorphous state of these complexes, meaning a transformation from crystalline to the amorphous state, and is strongly associated with the increased rate of dissolution rate seen in inclusion complexes [54].

#### 3.3.4. Dynamic Light Scattering (DLS) 

**Figure 6:**
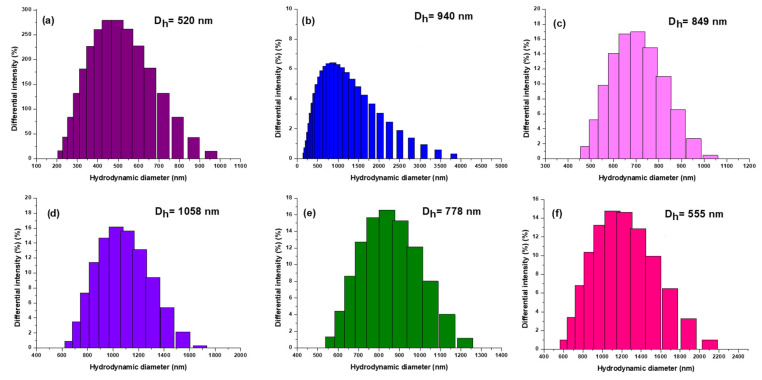
The hydrodynamic diameter (Dh) of 2HP-β-CD (**a**), NA-2HP-β-CD-HPMC (**b**), NA-2HP-β-CD (**c**), NAs-2HP-β-CD-HPMC (**d**), NA (**e**), and HPMC (**f**).

Figure 6 shows the results of the DLS analysis used to determine the size of the obtained complexes to illustrate their functionalization. According to the DLS histograms, dimensionally homogeneous assemblies appeared, with a notable increase in hydrodynamic diameter with different functionalizations.

The hydrodynamic diameter of 2HP-β-CD (Figure 6a) was initially observed to be 520 nm. Subsequently, NA-2HP-β-CD (Figure 6c) was successfully synthesized, and the functionalization was confirmed through a noticeable increase in the precursor’s diameter, which measured 849 nm. The process of functionalization resulted in the precursor NA-2HP-β-CD-HPMC (Figure 6b), which significantly increased the diameter to 940 nm. Similarly, the NAs-2HP-β-CD-HPMC (Figure 6d) showed a substantial increase in hydrodynamic diameter, reaching 1058 nm. This increase indicates that the drug has successfully formed a complex with the 2HP-β-CD structure, resulting in a larger and more complex molecular assembly. To provide further context, it is important to note that NA (Figure 6e), on its own, has a diameter of 778 nm, while HPMC (Figure 6f) has a diameter of 555 nm. This information underscores the stepwise functionalization process, in which each component contributes to the overall increase in diameter, ultimately leading to the formation of inclusion complexes with such hydrodynamic diameters. Another reason for the increase in diameter is the strong tendency for the agglomeration of the 2HP-β-CD as a consequence of the self-assembly in aqueous solutions to form aggregates with intermolecular linkage attributed to the OH groups located at the edges of the donut-shaped 2HP-β-CD molecules [74,75]. The complexes are ranked by diameter, as follows: NAs-2HP-β-CD-HPMC > NA-2HP-β-CD-HPMC > NA-2HP-β-CD. It is noteworthy that the particle size increased significantly after the formation of the ternary complexes. This increase is due to the high mobility of 2HP-β-CD complexes in solution and the possible interaction of non-ionic polymers with the outer surfaces of both CD and the inclusion complexes, which may enhance the interaction strength of the drug with 2HP-β-CD by forming large complex aggregates [76]. These results were confirmed by SEM micrographs. The diameter of the NAs-2HP-β-CD-HPMC complex was observed to be larger than that of other complexes, which can be attributed to enhanced attractive van der Waals forces resulting from the use of the NA salt. These intermolecular interactions cause the aggregation of NAs-2HP-β-CD-HPMC in the solution [74,77].

#### 3.3.5. Nuclear Magnetic Resonance Spectroscopy (NMR)

**Figure 7:**
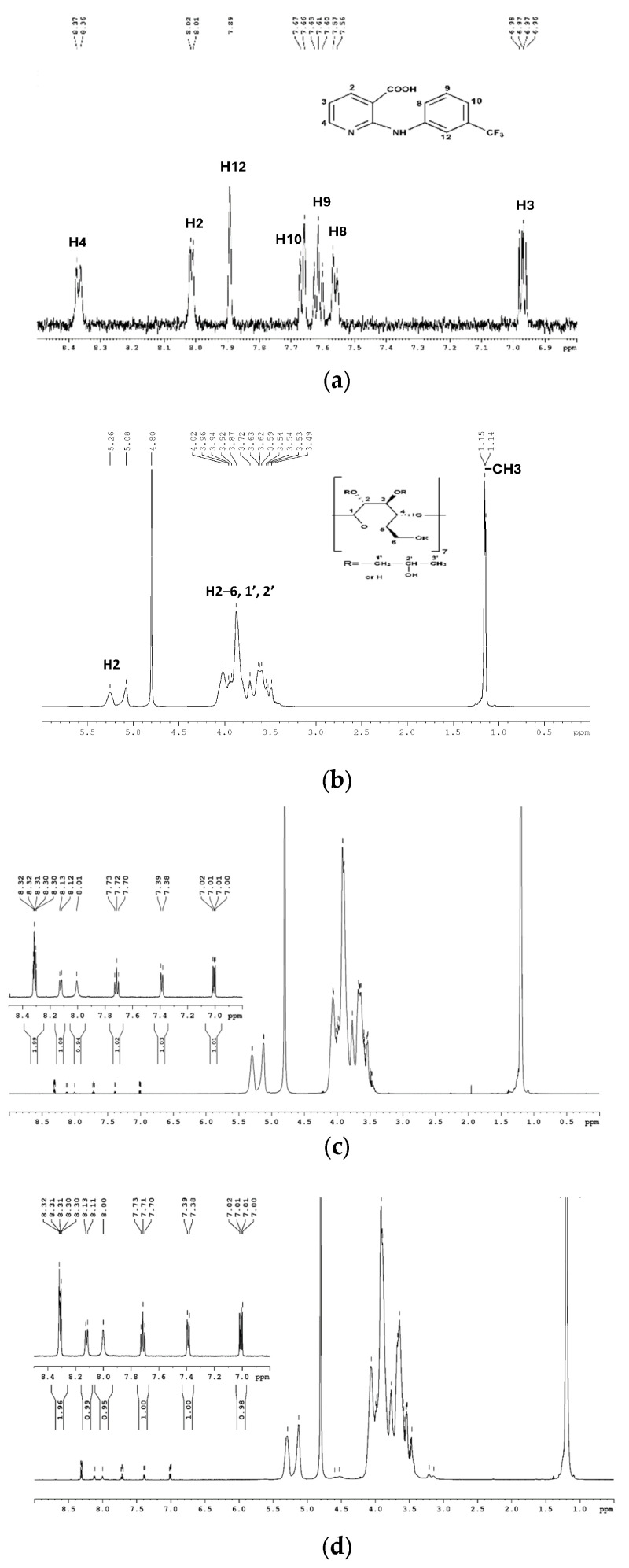

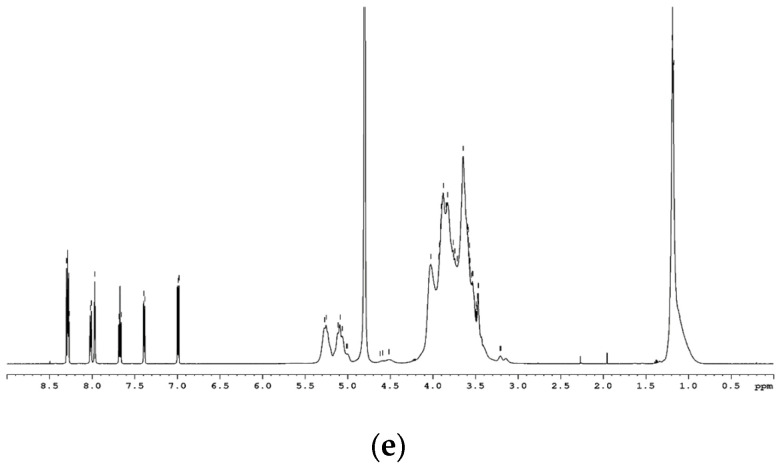
^1^H NMR spectra of NA (**a**), 2HP-β-CD (**b**), NA-2HP-β-CD (**c**), NA-2HP-β-CD-HPMC (**d**), and NAs-2HP-β-CD-HPMC (**e**).

Compared to previously described techniques, which cannot definitively distinguish between inclusion complex formation and adsorption phenomena or determine the structural conformation of molecules, NMR spectroscopy is a powerful tool for identifying the formation of an inclusion complex [78]. It provides direct evidence for the inclusion of a guest molecule in the CD cavity. If the guest molecule is included in the CD cavity, the H-3 and H-5 protons inside the cavity will be affected by the changed environment, resulting in characteristic chemical shift changes observable in the ¹H-NMR spectrum [79]. Figure 7 shows the ¹H-NMR spectra of NA, 2HP-β-CD, and the inclusion complexes. The proton signal assignments for NA (Figure 7a) and 2HP-β-CD (Figure 7b) were determined based on the relevant literature [35,80,81]. Thus, for 2HP-β-CD, most protons from glucose and propyl residues resonate in the interval of 3.48–4.01 ppm, with methyl groups resonating at 1.14–1.15 ppm and glucose H1 protons resonating at 5.08 and 5.26 ppm. NA has several sets of signals associated with the pyridine ring at 6.97 (H3), 8.01 (H2), and 8.37 (H4) ppm and with the phenyl ring at 7.56 (H8), 7.61 (H9), 7.66 (H10), and 7.89 (H12) ppm. The spectra of the inclusion complexes showed the proton signals of both NA and 2HP-β-CD, and their chemical shift values are presented in Table 3. Because, in the case of substituted CDs like 2HP-β-CD, the proton signals are severely overlapped and individual assignments are difficult to make, we followed the chemical shift variations of the guest protons (Table 3).

For the binary and ternary complexes, the protons located in both phenyl and pyridine rings showed significant variations, indicating that the NA molecule interacts with the interior of the 2HP-β-CD cavity [35]. 

**Figure 8:**
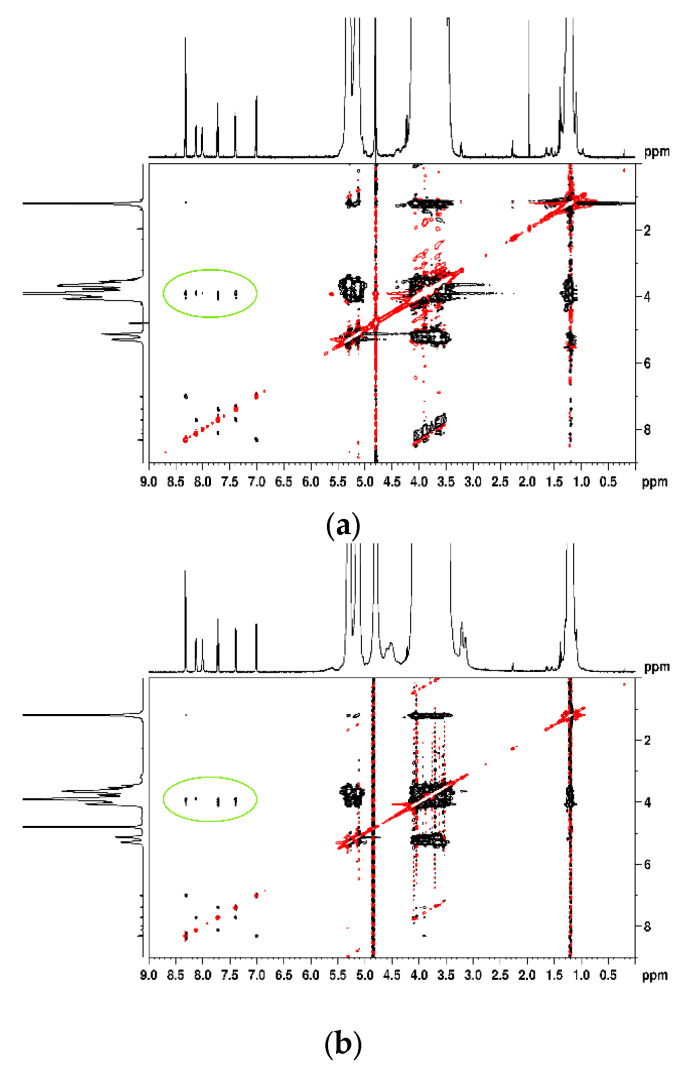

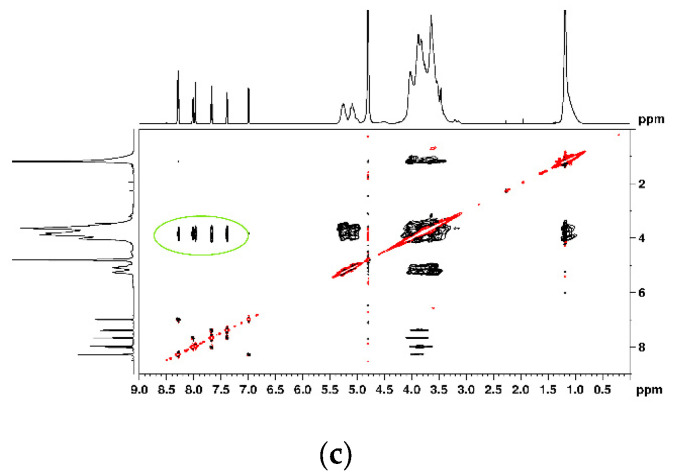
Two-dimensional ROSY spectra of NA-2HP-β-CD (**a**), NA-2HP-β-CD HPMC (**b**), and NAs-2HP-β-CD-HPMC (**c**).

Considering the dynamic nature between host and guest, these variations indicate the following two possible inclusion modes: one with the incorporation of trifluoromethylphenyl residue in the 2HP-β-CD cavity and the second one with a pyridine ring inside. These two inclusion modes are supported by the NOE cross-peaks visible in the 2D ROESY spectra (Figure 8). The figure emphasizes through-space interactions (circled in green) between protons from NA and 2HP-β-CD, situated less than 5 Å, which confirms the insertion of the guest molecule into the 2HP-β-CD cavity, providing additional conformational information about the synthesized binary and ternary inclusion complexes [54]. 

The molar ratios of the formed inclusion complexes were determined by analyzing the relative integrals of H1 protons from 2HP-β-CD and H9 protons from NA [82]. The analysis revealed a 1:24 molar ratio of NA to 2HP-β-CD for the NA-2HP-β-CD complex and a 1:12 ratio for the NA-2HP-β-CD-HPMC complex, while the NAs-2HP-β-CD-HPMC complex displayed a 1:1 stoichiometry ratio. Thus, only the NAs-2HP-β-CD-HPMC complex achieved the theoretical 1:1 host–guest molar ratio used during the initial preparation. This suggests that the NAs-2HP-β-CD-HPMC inclusion complex is more effective or stable [67]. These findings are consistent with the results of FT-IR, DSC, RDX, and the Kc and CE values obtained from the phase solubility study.

### 3.4. In Vitro Dissolution Studies

**Figure 9:**
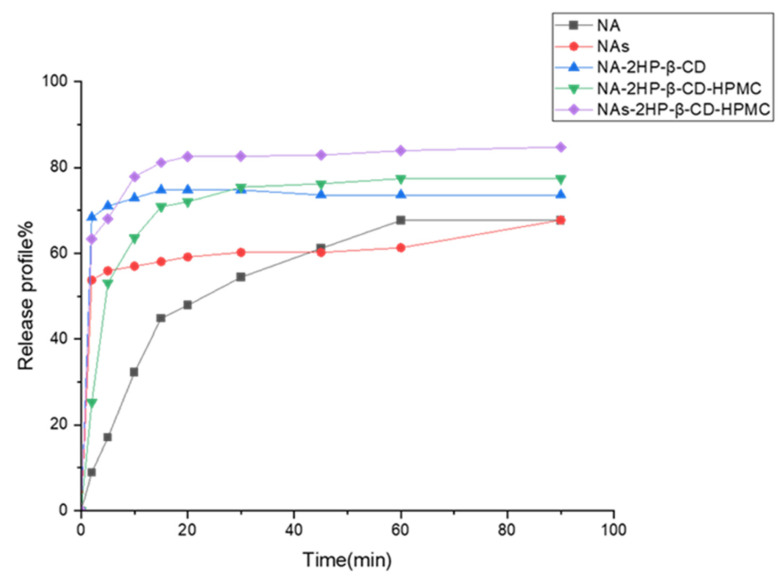
Dissolution profiles of NA, NAs, NA-2HP-β-CD, NA-2HP-β-CD-HPMC, and NAs-2HP-β-CD-HPMC (n = 3, coefficient of variation < 3%, error bars not shown for clarity).

This study assessed the dissolution rate of the drug and its complexes in simulated intestinal fluid as key parameters for understanding its potential bioavailability and evaluating the influence of complexation-induced physicochemical changes on the dissolution [83]. Figure 9 shows the dissolution profiles of NA, NAs, binary (NA-2HP-β-CD), and ternary (NA/NAs-2HP-β-CD-HPMC) inclusion complexes. An analysis of these profiles revealed that the binary and ternary inclusion complexes exhibited a significantly higher dissolution rate than pure NA and NAs. This increase in dissolution rate was attributed to the formation of inclusion complexes, which was confirmed by FT-IR, DSC, XRD, and ^1^H-NMR studies. This complexation induced several physicochemical changes to the drug molecule, such as amorphization, improvement of the wetting property, and the hydrophilicity of 2HP-β-CD [84,85]. For inclusion complexes, the cumulative drug release (%), after 20 min, was 82.53% for NAs-2HP-β-CD-HPMC, 72.02% for NA-2HP-β-CD-HPMC, and 74.74% for NA-2HP-β-CD-HPMC. In similar conditions, the dissolution rates for NA and NAs were only 47.9% and 59.14%, respectively. Notably, NAs-2HP-β-CD-HPMC showed the highest dissolution profile, probably due to its high complexation efficiency [27], as evidenced by phase solubility studies (Section 3.1). However, it is important to consider that the presence of HPMC can introduce additional factors affecting the release profile. Compared to the binary complex NA-2HP-β-CD, which reached its peak release rate at 15 min (74.74%), the ternary complexes with HPMC (NA-2HP-β-CD-HPMC and NAs-2HP-β-CD-HPMC) exhibited delayed peak dissolution rates, occurring at 60 min (77.39%) and 90 min (84.75%), respectively. This delay can be attributed to the swelling of the HPMC upon contact with the dissolution medium, which forms a hydrophilic layer and slows down the drug diffusion rate. This observation aligns with the findings of Soe et al. and Jug, M. and Becirevic-Lacan [86,87].

**Figure 10:**
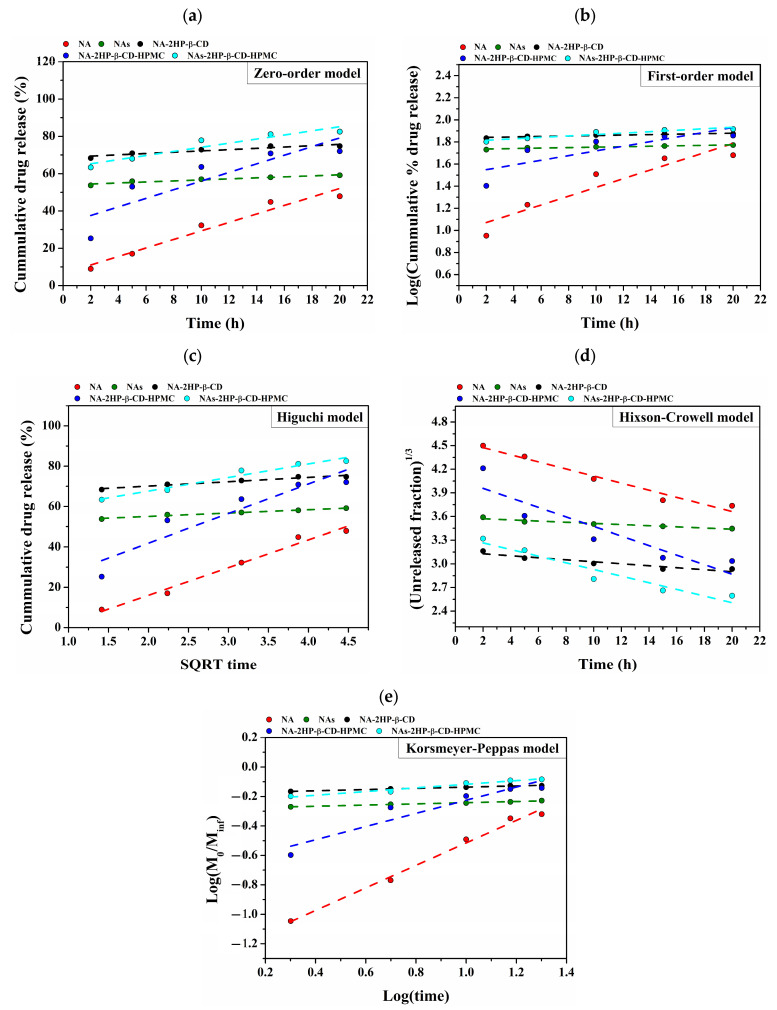
Linear fitting of the mathematical models applied for the drug release: zero-order model (**a**); first-order model (**b**); Higuchi model (**c**); Hixson–Crowell model (**d**); and Korsmeyer–Peppas model (**e**).

In order to obtain a more comprehensive understanding of the release mechanism, a variety of mathematical models, including the zero-order, first-order, Higuchi, Hixson–Crowell, and Korsmeyer–Peppas models, were fitted to the kinetic data. This study concentrated on the first part of the release curves (Figure 10). Different behaviors among the examined samples were identified by analyzing the correlation coefficients obtained from the mathematical models (Table 4). 

The release model that worked best for this type of system was selected from among mathematical models with correlation coefficients higher than 0.8. As a result, all mathematical models were adequate for the release data of the studied systems, producing correlation coefficients between 0.88 and 0.99; the only exception was the system NA-2HP-β-CD-HPMC, which had the lowest correlation coefficients between 0.67 and 0.90 on all applied models. Among the models that were examined, the Higuchi (Figure 10c) and Korsmeyer–Peppas (Figure 10e) models showed the best fit and had the greatest correlation coefficients of all the studied models.

Plotting the logarithm of cumulative percent drug release against the logarithm of time allowed us to establish the release exponent (n), which was used to investigate the release mechanism. The obtained values for the release exponent that are presented in Table 4 show that the NA and NA-2HP-β-CD-HPMC systems presented a non-Fickian diffusion mechanism, with values of the release exponent between 0.43 and 0.85; meanwhile, the systems NAs, NA-2HP-β-CD and NAs-2HP-β-CD-HPMC presented a Fickian diffusion mechanism, with values of the release exponent below 0.43 [88]. 

Moreover, the rate of drug release is indicated by the release constant (k), whose value is directly proportional to the diffusion constant (lower values suggest a slower release rate, while larger values indicate a quicker release rate) [89]. In our study, the slower release rate was observed in the case of the NA system, which presented the lowest value of the release constant. The other system presented similar values with the fastest release rate in the case of the NA-2HP-β-CD system, which is closely followed by NAs-2HP-β-CD-HPMC and NAs systems.

The Higuchi and Korsmeyer–Peppas models were found to be the most appropriate models for this kind of system based on an analysis of the applied release kinetics using mathematical models. The results obtained from this study confirm that the mechanism of drug release depends on the nature of the encapsulated drug, as well as the nature of the components that were used in the composition of the final system.

## 4. Conclusions

This study successfully developed and characterized the binary and ternary inclusion complexes of NA and its sodium salt with 2HP-β-CD and HPMC. A co-evaporation solvent and freeze-drying approach were employed for complexation. Extensive characterization, including a phase solubility study with an additional control group (NAs-2HP-β-CD) to evaluate the impact of using NAs alone, was conducted. This analysis determined the CE and Ks as key parameters of the formation efficiency of the complexes. Additionally, the drug encapsulation percentage was determined, and various physicochemical techniques (FT-IR, DSC, DLS, SEM, XRD, ^1^H-NMR, and 2D-ROESY) were utilized alongside dissolution studies. Physicochemical analyses confirmed complexation in all formulations, with the dissolution profiles of the complexes significantly exceeding those of pure NA and NAs. Notably, while the phase solubility study revealed that individual additions of HPMC or NAs offered modest improvements in either complexation efficiency or stability in the liquid state, their combined use in the NAs-2HP-β-CD-HPMC complex demonstrated a synergistic effect.

Among the studied complexes, the NAs-2HP-β-CD-HPMC formulation demonstrated the most promising results. It exhibited the highest CE and Ks values, the greatest drug loading, a 1:1 experimental molar ratio matching the theoretical value, substantial drug amorphization, and the most favorable dissolution profile. These findings suggest that the NAs-2HP-β-CD-HPMC complex holds significant potential as a candidate for improved NA delivery, potentially leading to enhanced bioavailability and therapeutic efficacy.

This study emphasizes the importance of optimized inclusion complexes for improved NA delivery. The comparative approach and diverse characterization techniques provided valuable insights that support further advancements in drug delivery systems, ultimately aiming to optimize therapeutic efficacy while minimizing side effects. Additionally, in vivo studies are necessary to confirm its clinical application potential. 

## Figures and Tables

**Table 1:** Phase solubility data of binary (NA/NAs-2HP-β-CD) and ternary (NA/NAs-2HP-β-CD-HPMC) systems.

System	S_0_ (M)	Slope ± SD	Ks (M^−1^) ± SD	CE ± SD	R^2^
NA-2HP-β-CD	0.00014	0.00786 ± 0.0004	55.29 ± 2.86	0.0079 ± 0.0004	0.98807
NA-2HP-β-CD-HPMC	0.00014	0.00868 ± 0.0007	62.54 ± 5.00	0.0087 ± 0.0007	0.96595
NAs-2HP-β-CD	0.00810	0.16155 ± 0.0209	23.79 ± 2.63	0.19 ± 0.021	0.92233
NAs-2HP-β-CD-HPMC	0.00810	0.54571 ± 0.0099	148.30 ± 1.23	1.2 ± 0.01	0.99934

Data were presented as mean ± SD, n = 3.

**Table 2:** Drug content (%) and the amount of the drug in binary (NA-2HP-β-CD) and ternary (NA/NAs-2HP-β-CD-HPMC) systems.

Complex	Drug Content (%) ± SD	Drug (mg) in 10 mg Complex ± SD
NA-2HP-β-CD	0.67% ± 0.03%	0.067 ± 1.91
NA-2HP-β-CD-HPMC	1.29% ± 0.04%	0.129 ± 1.76
NAs-2HP-β-CD-HPMC	12.40% ± 0.08%	1.24 ± 2.65

Data were presented as mean ± SD, n = 3.

**Table 3:** ^1^H NMR chemical shift change (ppm) data of NA protons in the free state and inclusion complexes (Δδ = δcomplex − δfree).

Proton	δ (Free)	δ (NA-2HP-β-CD)	Δδ	δ (NA-2HP-β-CD-HPMC)	Δδ	δ (NAs-2HP-β-CD-HPMC)	Δδ
				NA			
H-2	8.01	8.30–8.32	0.29–0.31	8.30–8.32	0.29–0.31	8.27–8.30	0.26–0.29
H-3	6.97	7.01	0.04	7.01	0.04	6.99	0.02
H-4	8.37	8.30–8.32	−0.07–−0.05	8.30–8.32	−0.07–−0.05	8.27–8.30	−0.1–−0.07
H-8	7.56	7.39	−0.17	7.39	−0.17	7.39	−0.17
H-9	7.61	7.72	0.11	7.71	0.10	7.67	0.06
H-10	7.66	8.12	0.46	8.12	0.46	8.02	0.36
H-12	7.89	8.01	0.12	8.00	0.11	7.97	0.08

**Table 4:** The data obtained by applying various mathematical models to analyze the release kinetics of free drug during the initial phase (2–20 h) of the release process.

Sample	Zero-Order		First-Order		Higuchi		Hixson–Crowell		Korsmeyer–Peppas		
	R^2^	K_0_	R^2^	K_t_	R^2^	K_H_	R^2^	k	R^2^	k	n
NA	0.96	2.28	0.88	−0.09	0.98	13.71	0.97	−0.045	0.98	0.06	0.705
NAs	0.94	0.27	0.93	−0.005	0.98	1.55	0.94	−0.007	0.99	0.52	0.041
NA-2HP-β-CD	0.89	0.35	0.88	−0.005	0.96	2.16	0.90	−0.013	0.99	0.66	0.041
NA-2HP-β-CD-HPMC	0.77	2.31	0.67	−0.05	0.87	14.64	0.83	−0.060	0.90	0.21	0.446
NAs-2HP-β-CD-HPMC	0.90	1.10	0.89	−0.01	0.96	6.74	0.93	−0.042	0.97	0.57	0.124

where R^2^ is the correlation coefficient, K is the proportionality constant, and n is the release exponent.

## Data Availability

The original contributions presented in the study are included in the article, further inquiries can be directed to the corresponding author.

## References

[1] Venkatesh S., Lipper R.A. (2000). Role of the development scientist in compound lead selection and optimization. J. Pharm. Sci..

[2] Pandey S.P., Shukla T., Dhote V.K., Mishra D.K., Maheshwari R., Tekade R.K., Tekade R.K. (2019). Use of polymers in controlled release of active agents. Advances in Pharmaceutical Product Development and Research, Basic Fundamentals of Drug Delivery.

[3] Ambrus R., Radacsi N., Szunyogh T., van der Heijden A., ter Horst J., Szabó-Révész P. (2013). Analysis of submicron-sized niflumic acid crystals prepared by electrospray crystallization. J. Pharm. Biomed. Anal..

[4] Szunyogh T., Ambrus R., Szabó-Révész P. (2013). Nanonization of niflumic acid by co-grinding. Adv. Nanoparticles.

[5] Bag P.P., Reddy C.M. (2012). Screening and selective preparation of polymorphs by fast evaporation method: A case study of aspirin, anthranilic acid, and niflumic acid. Cryst. Growth Des..

[6] Assas N., Elbahri Z., Baitiche M., Djerboua F. (2019). Effects of some process parameters on the niflumic acid controlled release polymeric microspheres: Optimization using designs of experiments. Asia Pac. J. Chem. Eng..

[7] Acebedo-Martínez F.J., Alarcón-Payer C., Frontera A., Barbas R., Prohens R., Di Crisci M., Domínguez-Martín A., Gómez-Morales J., Choquesillo-Lazarte D. (2021). Novel polymorphic cocrystals of the non-steroidal anti-inflammatory drug niflumic acid: Expanding the pharmaceutical landscape. Pharmaceutics.

[8] Bashir S., Zafar N., Lebaz N., Mahmood A., Elaissari A. (2020). Hydroxypropyl methylcellulose-based hydrogel copolymeric for controlled delivery of galantamine hydrobromide in dementia. Processes.

[9] Lakkakula J., Macedo Krause R.W., Barage S., Joshi A., Patil S., Khan A.A., Roy A. (2024). Exploring oral drug delivery: In vitro release and mathematical modeling of hydrophobic drug (Na-L-thyroxine) and its cyclodextrin inclusion complex in chitosan microparticles. Int. J. Biol. Macromol..

[10] Muankaew C., Loftsson T. (2018). Cyclodextrin-based formulations: A non-invasive platform for targeted drug delivery. Basic Clin. Pharmacol. Toxicol..

[11] Subramanian E., Anitha G., Selvam M.K., Badusha M.I.A. (2005). Influence of β-cyclodextrin as an encapsule and as an inclusion complex dopant on conducting polyaniline. B Mater. Sci..

[12] Sharma N., Baldi A. (2016). Exploring versatile applications of cyclodextrins: An overview. Drug Deliv..

[13] Singh J., Dhamija I., Meehenian H., Kumar N., Simran S., Muskan M., Verma M.L., Kumar S. (2022). Chronicle updates in cyclodextrin-based carriers for drug delivery. Bull. Natl. Res. Cent..

[14] Sung Y.K., Kim S.W. (2020). Recent advances in polymeric drug delivery systems. Biomater. Res..

[15] Gould S., Scott R.C. (2005). 2-Hydroxypropyl-β-cyclodextrin (HP-β-CD): A toxicology review. Food Chem. Toxicol..

[16] Nie C., Liang Q., Gao Q. (2024). Preparation of Eudragit S100-pullulan/hydroxypropyl-β-cyclodextrin complex-Eudragit S100 multilayer nanofiber film for resveratrol colon delivery. Int. J. Biol. Macromol..

[17] Jambhekar S., Casella R., Maher T. (2004). The physicochemical characteristics and bioavailability of indomethacin from β-cyclodextrin, hydroxyethyl-β-cyclodextrin, and hydroxypropyl-β-cyclodextrin complexes. Int. J. Pharm..

[18] Del Valle E.M. (2004). Cyclodextrins and their uses: A review. Process Biochem..

[19] Spulber M., Pinteala M., Fifere A., Harabagiu V., Simionescu B.C. (2008). Inclusion complexes of 5-flucytosine with β-cyclodextrin and hydroxypropyl-β-cyclodextrin: Characterization in aqueous solution and in solid state. J. Incl. Phenom. Macrocycl. Chem..

[20] Loftsson T., Sigurdsson H.H., Másson M., Schipper N. (2004). Preparation of solid drug/cyclodextrin complexes of acidic and basic drugs. Pharmazie.

[21] Loftsson T., Hreinsdóttir D., Másson M. (2007). The complexation efficiency. J. Incl. Phenom. Macrocycl. Chem..

[22] Jacob S., Nair A.B. (2018). Cyclodextrin complexes: Perspective from drug delivery and formulation. Drug Develop. Res..

[23] Loh G.O.K., Tan Y.T.F., Peh K.K. (2014). Effect of HPMC concentration on β-cyclodextrin solubilization of norfloxacin. Carbohydr. Polym..

[24] Loftsson T., Másson M. (2004). The effects of water-soluble polymers on cyclodextrins and cyclodextrin solubilization of drugs. J. Drug Deliv. Sci. Technol..

[25] Moussa E., Siepmann F., Flament M.P., Benzine Y., Penz F., Siepmann J., Karrout Y. (2019). Controlled release tablets based on HPMC:lactose blends. J. Drug Deliv. Sci. Technol..

[26] Shamshad Malik N., Ahmad M., Minhas M.U. (2017). Cross-linked β-cyclodextrin and carboxymethyl cellulose hydrogels for controlled drug delivery of acyclovir. PLoS ONE.

[27] Li Z., Johnson L.M., Ricarte R.G., Yao L.J., Hillmyer M.A., Bates F.S., Lodge T.P. (2017). Enhanced performance of blended polymer excipients in delivering a hydrophobic drug through the synergistic action of micelles and HPMCAS. Langmuir.

[28] Romero S., Escalera B., Bustamante P. (1999). Solubility behavior of polymorphs I and II of mefenamic acid in solvent mixtures. Int. J. Pharm..

[29] Szunyogh T., Ambrus R., Szabó-Révész P. (2012). Formation of niflumic acid particle size by solvent diffusion and solvent evaporation as precipitation methods. J. Drug Deliv. Sci. Technol..

[30] Kawabata Y., Wada K., Nakatani M., Yamada S., Onoue S. (2011). Formulation design for poorly water-soluble drugs based on biopharmaceutics classification system: Basic approaches and practical applications. Int. J. Pharm..

[31] Jang D.J., Park J.-S., Ko H.-R., Jee J.-P., Kim J.-K., Kim S.T., Kim C.-K. (2005). Simultaneous determination of niflumic acid and its prodrug, talniflumate in human plasma by high performance liquid chromatography. Biomed. Chromatogr..

[32] Kang W., Kim K. (2009). Determination of talniflumate and niflumic acid in human plasma by liquid chromatography-tandem mass spectrometry. Anal. Sci..

[33] Lervolino M., Quaglia F., Miro A., Calignano A., Cappello B. (2004). Reduced gastric toxicity of niflumic acid/cyclodextrin systems. J. Drug Deliv. Sci. Technol..

[34] Terekhova I.V., Volkova T.V., Perlovich G.L. (2006). Experimental analysis of complex formation of niflumic acid with β-Cyclodextrins. J. Incl. Phenom. Macrocycl. Chem..

[35] Bogdan M., Caira M.R., Farcas S.I. (2002). Inclusion of the niflumic acid anion in β-cyclodextrin: A solution NMR and X-ray structural investigation. Supramol. Chem..

[36] Kata M., Ambrus R., Aigner Z. (2002). Preparation and investigation of inclusion complexes containing nifluminic acid and cyclodextrins. J. Incl. Phenom. Macrocycl. Chem..

[37] Redenti E., Szente L., Szejtli J. (2001). Cyclodextrin complexes of salts of acidic drugs. Thermodynamic properties, structural features, and pharmaceutical applications. J. Pharm. Sci..

[38] Loftsson T., Brewster M.E. (2012). Cyclodextrins as functional excipients: Methods to enhance complexation efficiency. J. Pharm. Sci..

[39] Higuchi T. (1965). Phase-solubility techniques. Adv. Anal. Chem. Instr..

[40] Jansook P., Loftsson T. (2009). CDs as solubilizers: Effects of excipients and competing drugs. Int. J. Pharm..

[41] Loftsson T., Hreinsdóttir D., Masson M. (2005). Evaluation of cyclodextrin solubilization of drugs. Int. J. Pharm..

[42] Yurtdaş G., Demirel M., Genç L. (2011). Inclusion complexes of fluconazole with β-cyclodextrin: Physicochemical characterization and in vitro evaluation of its formulation. J. Incl. Phenom. Macrocycl. Chem..

[43] Agüeros M., Ruiz-Gatón L., Vauthier C., Bouchemal K., Espuelas S., Ponchel G., Irache J.M. (2009). Combined hydroxypropyl-β-cyclodextrin and poly (anhydride) nanoparticles improve the oral permeability of paclitaxel. Eur. J. Pharm. Sci..

[44] Loftsson T., Brewster M.E. (1996). Pharmaceutical applications of cyclodextrins. 1. Drug solubilization and stabilization. J. Pharm. Sci..

[45] Melnik M., Maca’skova L., Holloway C.E. (1993). Spectroscopic and magnetic properties of copper (II) niflumates. J. Coord. Chem..

[46] Gholibegloo E., Mortezazadeh T., Salehian F., Ramazani A., Amanlou M., Khoobi M. (2019). Improved curcumin loading, release, solubility and toxicity by tuning the molar ratio of cross-linker to β-cyclodextrin. Carbohydr. Polym..

[47] United States Pharmacopeial Convention (2010). USP 33 NF 28: United States Pharmacopeia [and] National Formulary. Reissue. Supplement 2.a.

[48] Craciun B.-F., Sandu I.A., Peptanariu D., Pinteala M. (2023). Novel nanotherapeutic systems based on pegylated squalene micelles for enhanced in vitro activity of methotrexate and cytarabine. Polymers.

[49] Sid D., Baitiche M., Elbahri Z., Djerboua F., Boutahala M., Bouaziz Z., Le Borgne M. (2021). Solubility enhancement of mefenamic acid by inclusion complex with β-cyclodextrin: In silico modelling, formulation, characterisation, and in vitro studies. J. Enzyme Inhib. Med. Chem..

[50] Rudrangi S.R.S., Bhomia R., Trivedi V., Vine G.J., Mitchell J.C., Alexander B.D., Wicks S.R. (2015). Influence of the preparation method on the physicochemical properties of indomethacin and methyl-β-cyclodextrin complexes. Int. J. Pharm..

[51] Hirlekar R.S., Sonawane S.N., Kadam V.J. (2009). Studies on the effect of water-soluble polymers on drug–cyclodextrin complex solubility. AAPS Pharm. Sci. Tech..

[52] Kim Y., Oksanen D.A., Massefski W., Blake J.F., Duffy E.M., Chrunyk B. (1998). Inclusion complexation of ziprasidone mesylate with beta-cyclodextrin sulfobutyl ether. J. Pharm. Sci..

[53] Rama A.C.R., Veiga F., Figueiredo I.V., Sousa A., Caramona M. (2005). Aspectos biofarmacêuticos da formulação de medicamentos para neonatos: Fundamentos da complexação de indometacina com hidroxipropil-beta-ciclodextrina para tratamento oral do fechamento do canal arterial. Braz. J. Pharm. Sci..

[54] Goh S.Q., Adnan R., Rahim N.Y. (2022). Mini review on synthesis and characterization methods for ternary inclusion complexes of cyclodextrins with drugs. Malays. J. Chem..

[55] Balci K., Akkaya Y., Akyuz S. (2010). An experimental and theoretical vibrational spectroscopic study on niflumic acid, a non-steroidal anti-inflammatory drug. Vib. Spectrosc..

[56] Paduraru O.M., Bosinceanu A., Tantaru G., Vasile C. (2013). Effect of Hydroxypropyl-β-Cyclodextrin on the Solubility of an Antiarrhythmic. Agent. Ind. Eng. Chem. Res..

[57] Ahad A., Bin Jardan Y.A., Raish M., Al-Mohizea A.M., Al-Jenoobi F.I. (2022). Ternary inclusion complex of sinapic acid with hydroxypropyl-β-cyclodextrin and hydrophilic polymer prepared by microwave. Processes.

[58] Sherje A.P., Patel F., Murahari M., Suvarna V., Patel K. (2018). Study on effect of L-arginine on solubility and dissolution of Zaltoprofen: Preparation and characterization of binary and ternary cyclodextrin inclusion complexes. Chem. Phys. Lett..

[59] da Silva Mourao L.C., Ribeiro Batista D.R.M., Braga Honorato S., Ayala A.P., de Alencar Morais W., Barbosa E.G., Nervo Raffin F., Accioly de Lima e Moura T.F. (2016). Effect of hydroxypropyl methylcellulose on beta cyclodextrin complexation of praziquantel in solution and in solid state. J. Incl. Phenom. Macrocycl. Chem..

[60] Cozma V., Rosca I., Radulescu L., Martu C., Nastasa V., Varganici C.-D., Ursu E.-L., Doroftei F., Pinteala M., Racles C. (2021). Antibacterial polysiloxane polymers and coatings for cochlear implants. Molecules.

[61] Uritu C.M., Varganici C.D., Ursu L., Coroaba A., Nicolescu A., Dascalu A.I., Peptanariu D., Stan D., Constantinescu C.A., Simion V. (2015). Hybrid fullerene conjugates as vectors for DNA cell-delivery. J. Mater. Chem. B.

[62] Varganici C.-D., Ursache O., Gaina C., Gaina V., Simionescu B.C. (2013). Studies on new hybrid materials prepared by both Diels–Alder and Michael addition reactions. J. Therm. Anal. Calorim..

[63] Ştiubianu G., Soroceanu A., Varganici C.-D., Tugui C., Cazacu M. (2016). Dielectric elastomers based on silicones filled with transitional metal complexes. Compos. B Eng..

[64] Jadhav P., Pore Y. (2017). Physicochemical, thermodynamic and analytical studies on binary and ternary inclusion complexes of bosentan with hydroxypropyl-β-cyclodextrin. Bull. Fac. Pharm. Cairo Univ..

[65] Minea B., Marangoci N., Peptanariu D., Rosca I., Nastasa V., Corciova A., Varganici C., Nicolescu A., Fifere A., Neamtu A. (2016). Inclusion complexes of propiconazole nitrate with substituted β-cyclodextrins: The synthesis and in silico and in vitro assessment of their antifungal properties. New J. Chem..

[66] Mura P. (2015). Analytical techniques for characterization of cyclodextrin complexes in the solid state: A review. J. Pharm. Biomed. Anal..

[67] Grebogi I.H., Tibola A.P.O.V., Barison A., Grandizoli C.W.P.S., Ferraz H.G., Rodrigues L.N.C. (2012). Binary and ternary inclusion complexes of dapsone in cyclodextrins and polymers: Preparation, characterization and evaluation. J. Incl. Phenom. Macrocycl. Chem..

[68] Corciova A., Ciobanu C., Poiata A., Nicolescu A., Drobota M., Varganici C.D., Pinteala T., Fifere A., Marangoci N., Mircea C. (2014). Inclusion complexes of hesperidin with hydroxypropyl-β-cyclodextrin. Physico-chemical characterization and biological assessment. Dig. J. Nanomater. Biostruct..

[69] Vasincu I.M., Apotrosoaei M., Lupascu F., Iacob A.-T., Giusca S.-E., Caruntu I.-D., Marangoci N.-L., Petrovici A.R., Stanciu G.D., Tamba B.-I. (2023). Complexes of ibuprofen thiazolidin-4-one derivatives with β-cyclodextrin: Characterization and in vivo release profile and biological evaluation. Pharmaceutics.

[70] Varganici C.-D., Marangoci N., Rosu L., Barbu-Mic C., Rosu D., Pinteala M., Simionescu B.C. (2015). TGA/DTA–FTIR–MS coupling as analytical tool for confirming inclusion complexes occurrence in supramolecular host–guest architectures. J. Anal. Appl. Pyrolysis.

[71] Tundisi L., Mostaço G.B., Carricondo P.C., Petri D.F.S. (2021). Hydroxypropyl methylcellulose: Physico-chemical properties and ocular drug delivery formulations. Eur. J. Pharm. Sci..

[72] Hładon T., Cwiertnia B. (1994). Physical and chemical interactions between cellulose ethers and β-cyclodextrins. Pharmazie.

[73] Radacsi N., Giapis K.P., Ovari G., Szabó-Révész P., Ambrus R. (2019). Electrospun nanofiber-based niflumic acid capsules with superior physicochemical properties. J. Pharm. Biomed. Anal..

[74] Ryzhakov A., Thi T.D., Stappaerts J., Bertoletti L., Kimpe K., Sá Couto A.R., Saokham P., Van denMooter G., Augustijns P., Somsen G.W. (2016). Self-assembly of cyclodextrins and their complexes in aqueous solutions. J. Pharm. Sci..

[75] Messner M., Kurkov S.V., Maraver Palazón M., Álvarez Fernández B., Brewster M.E., Loftsson T. (2011). Self-assembly of cyclodextrin complexes: Effect of temperature, agitation and media composition on aggregation. Int. J. Pharm..

[76] He J., Chen H.-Y., Chen H., Wang B., Guo F., Zheng Z.-P. (2022). Characterization, stability, and antibrowning effects of oxyresveratrol cyclodextrin complexes combined use of hydroxypropyl methylcellulose. Foods.

[77] Kotronia M., Kavetsou E., Loupassaki S., Kikionis S., Vouyiouka S., Detsi A. (2017). Encapsulation of oregano (*Origanum onites* L.) essential oil in β-cyclodextrin (β-CD): Synthesis and characterization of the inclusion complexes. Bioengineering.

[78] Djedaïni F., Perly B. (1991). Nuclear magnetic resonance investigation of the stoichiometries in β-cyclodextrin: Steroid inclusion complexes. J. Pharm. Sci..

[79] Inoue Y. (1993). NMR studies of the structure and properties of cyclodextrins and their inclusion complexes. Annu. Rep. NMR Spectrosc..

[80] Soares L.A., Vasconcelos Borges Leal A.F., Fernandes Fraceto L., Elaine R.M., Sabioni Resck I., Massuo J.K., de Sousa Gil E., de Sousa A.R., da Cunha L.C., Rezende K.R. (2009). Host–guest system of 4-nerolidylcatechol in 2-hydroxypropyl-β-cyclodextrin: Preparation, characterization and molecular modeling. J. Incl. Phenom. Macrocycl. Chem..

[81] Van Axel Castelli V., Trivieri G., Zucchelli I., Brambilla L., Barbuzzi T., Castiglioni C., Paci M., Zerbi G., Zanol M. (2008). Characterisation of an inclusion complex between cladribine and 2-hydroxypropyl-betacyclodextrin. J. Pharm. Sci..

[82] Chen M., Diao G., Zhang E. (2006). Study of inclusion complex of β-cyclodextrin and nitrobenzene. Chemosphere.

[83] Herman J., Remon J.P., Lefebvre R., Bogaert M., Klinger G.H., Schwartz J.B. (1988). The dissolution rate and bioavailability of hydrochlorothiazide in pellet formulations. J. Pharm. Pharmacol..

[84] Chen C.-Y., Chen F.-A., Wu A.-B., Hsu H.-C., Kang J.-J., Cheng H.-W. (1996). Effect of hydroxypropyl-β-cyclodextrin on the solubility, photostability and in-vitro permeability of alkannin/shikonin enantiomers. Int. J. Pharm..

[85] Shah M., Pore Y., Dhawale S., Burade K., Kuchekar B. (2013). Physicochemical characterization of spray dried ternary micro-complexes of cefuroxime axetil with hydroxypropyl-β-cyclodextrin. J. Incl. Phenom. Macrocycl. Chem..

[86] Soe H.M.H., Chamni S., Mahalapbutr P., Kongtaworn N., Rungrotmongkol T., Jansook P. (2020). The investigation of binary and ternary sulfobutylether-β-cyclodextrin inclusion complexes with asiaticoside in solution and in solid state. Carbohyd. Res..

[87] Jug M., Bećirević-Laćan M. (2004). Multicomponent complexes of piroxicam with cyclodextrins and hydroxypropyl methylcellulose. Drug Dev. Ind. Pharm..

[88] Korsmeyer R.W., Gurny R., Doelker E., Buri P., Peppas N.A. (1983). Mechanisms of solute release from porous hydrophilic polymers. Int. J. Pharm..

[89] Unagolla J.M., Jayasuriya A.C. (2018). Drug transport mechanisms and in vitro release kinetics of vancomycin encapsulated chitosan-alginate polyelectrolyte microparticles as a controlled drug delivery system. Eur. J. Pharm. Sci..

